# Response in Growth, Scute Development, and Whole-Body Ion Composition of *Acipenser fulvescens* Reared in Water of Differing Chemistries

**DOI:** 10.3390/ani11051419

**Published:** 2021-05-15

**Authors:** Janet Genz, Rachael N. Hicks

**Affiliations:** Department of Mathematics, Science and Technology, University of West Georgia, Carrollton, GA 30118, USA

**Keywords:** calcium, Lake Sturgeon, larval development, zinc

## Abstract

**Simple Summary:**

In fishes, the relationship between environmental concentration of ions and internal availability is closely linked. Environmental ion limitation can have substantial effects on early life stages and growth and potentially reduce development of predatory defenses. This study examined whether different environmental ion levels affect the growth and development of protective structures in a species of conservation interest, the Lake Sturgeon (*Acipenser fulvescens*). We hatched sturgeon eggs in water from two sources varying in ionic composition: the Warm Springs National Fish Hatchery and the Coosa River. Each water type had a stable pH (7.3 ± 0.09) and temperature (15 ± 1 °C) throughout the experiment, and the environmental concentrations of calcium, magnesium, potassium, sodium, and zinc were quantified for collected water samples. These same ions were also quantified in the tissue of the larval fish during the first eight weeks of development post-hatch. Results indicate that the ion content of larval fish mirrors the environmental differences, and that the growth rate is slower in natural river water, which has lower levels of calcium (14.0 ± 0.24 mg/L) and higher amounts of zinc (0.13 ± 0.02 mg/L). Understanding environmental impacts on growth rate and development of defensive structures is important to re-establishing a self-recruiting *A. fulvescens* population in Georgia waterways.

**Abstract:**

In fishes, environmental ion availability can have substantial effects on growth and development. This study examined the development of Lake Sturgeon in response to the varying environmental ion availability that they experience as part of a conservation stocking program. We reared sturgeon in natural water from the Coosa River, which had higher concentrations of Mg^2+^, Na^+^, and Zn^2+^ than standard hatchery conditions, while [Ca^2+^] at the Warm Springs National Fish Hatchery was 2× higher than in the Coosa River. Eggs were hatched in each water type and the larvae were sampled at time points before and after yolk absorption during the first 8 weeks of development. Total length and weight in WSNFH larvae were significantly higher than larvae in Coosa River water starting at 8 dph, indicating that growth was dependent on the different environmental ion levels. Concentrations of the ions of interest were also determined for whole-body acid digests of the exposed Lake Sturgeon. We found that Lake Sturgeon reared in Coosa River water had significantly higher magnesium and zinc than Lake Sturgeon reared in WSNFH water (*p* < 0.05), while calcium was significantly higher in WSNFH than Coosa River water. This difference shows that different environmental ion concentrations also impact the overall development of larval Lake Sturgeon.

## 1. Introduction

Lake Sturgeon, *Acipenser fulvescens,* are a species ancestral to the teleost fishes, in the division Osteichthyes (bony fishes), and part of the class Actinopterygii (ray-finned fishes). Lake Sturgeon have a rich history in America; until the 1870s, they were considered a “nuisance” with little to no value. Not much care was put into the conservation of Lake Sturgeon populations and when they were caught by fishermen, they were killed, thrown back, or left on the river/lake banks [1]. It was not until they were used for caviar that Lake Sturgeon were considered of value, and this change contributed to additional population declines. Habitat disturbance and pollution in lakes further decreased the population levels [2,3]. Lake Sturgeon are one of the largest freshwater fish, and have one of the longest life spans. The oldest specimen recorded was caught in 1952 and was estimated to be 152 years old [1,4]. Female Lake Sturgeon reach sexual maturity in 14 to 24 years and males at approximately 14 to 16 years old [5]. It was not until the mid-to-late 20th century that people started to realize that populations were declining at an alarming rate and research toward their conservation began in earnest. In 1986, Lake Sturgeon were listed as vulnerable on the International Union for Conservation of Nature (IUCN) red list due to populations declining. With the help of management and protection plans, in 2004 Lake Sturgeon were listed as a species of least concern by IUCN, and populations are increasing [1]. The northernmost range of Lake Sturgeon is Hudson Bay in Canada, while the southernmost populations of Lake Sturgeon in North America are found in the Coosa River in Georgia and Alabama. Populations have been declining in the Southern United States region [6,7] and to re-establish the extirpated population, the Georgia Department of Natural Resources (GA-DNR) established a conservation stocking program for Lake Sturgeon into the Coosa River in 2002.

It is important to observe what differences in development may exist between the river where Lake Sturgeon naturally occur and the hatchery where Lake Sturgeon are reared for stocking. There are some important similarities and differences between the Warm Springs National Fish Hatchery (WSNFH) and Coosa River water. In the Coosa River water, magnesium, zinc, and sodium are significantly higher (Table 1) [8]. In WSNFH water, the levels of calcium are almost double the concentration found in Coosa River water. Potassium levels are almost the same between the two systems (Coosa River: 1.59 ± 0.06 vs. WSNFH: 1.62 ± 0.16 mg/L). This study used water collected from the Coosa River to see what effect it will have on Lake Sturgeon when they are developing in order to provide insight into how stocked juveniles may differ from naturally recruited young of the year. We also measured ions in the water from the Warm Springs National Fish Hatchery because young-of-year Lake Sturgeon are being kept and grown annually by that facility so we could compare the water at WSNFH to the Coosa River water. Knowing how Lake Sturgeon develop in the wild could help conservation projects in the future.

Lake Sturgeon can absorb calcium, magnesium, zinc, potassium, and sodium in more ways than simply ingesting them through their food. They can also intake calcium at the gills by mitochondria-rich cells (MRC), also known as chloride cells or ionocytes [9,10,11]. In teleost fishes, the gills are where most of the calcium is absorbed [10]. In larval fish, ions can be also absorbed through the skin [11,12]. For all the ions (calcium, magnesium, zinc, potassium, and sodium), where they are absorbed depends on the amounts of ions in the water. If the water ion concentrations are low, they may absorb more from the food to make up for the low levels [13]. It is estimated that about 30% of calcium is absorbed through the intestines in fishes such as *Gadus morhua* [9]. There is not a lot known regarding the natural diet of exogenously feeding larval Lake Sturgeon. It is believed that young sturgeon eat small invertebrates and are omnivorous [6]. This study focused on the calcium uptake from the water and controlled dietary ion input in an attempt to isolate effects that reflect only branchial, rather than intestinal, absorption. We also focused on zinc because this heavy metal has been found at sub-lethal levels in WSNFH water and Coosa River water and accumulates in Lake Sturgeon tissues. A buildup of zinc in the body of a Lake Sturgeon that absorbs or consumes it may cause overall growth to slow over time, as is the case in other bony fishes [14]. It has also been shown that a decrease in the calcium levels also decreases the zinc levels [15].

Early in a Lake Sturgeons’ life, they have a high requirement for calcium to support their development [16], but the freshwater lakes and rivers in which they are found typically do not have high levels of calcium [13]. An important question is how do Lake Sturgeon in the Southeastern United States get enough calcium to grow and develop normally? Available information on calcium regulation in larval and juvenile Lake Sturgeon is very limited, and we still do not fully understand the mechanisms driving uptake of calcium in Lake Sturgeon [17]. While in the pre-larval stage, Lake Sturgeon obtain the calcium they need from their yolk and subsequently rely on branchial and intestinal transport, similar to other fishes [11,18]. Larval Lake Sturgeon are able to increase their calcium absorption rates if their environments have low levels to try and combat low levels of calcium, as is the case for most bony fishes [11]. Since Lake Sturgeon are not typically found in water that is high in calcium, we are interested in how naturally recruited Lake Sturgeon larvae are surviving with these lower levels of environmental calcium. They might be utilizing these uptake mechanisms to obtain calcium from their environment and then storing the calcium in body tissues. Understanding the storage of calcium in developing Lake Sturgeon can help the population in the future, since calcium is important to their development and growth.

A possible storage reservoir for absorbed calcium may be the bony scutes developed in juvenile Lake Sturgeon. Sturgeon scutes are a form of ganoid scales, with five rows arranged longitudinally along the dorsal and lateral lines of Lake Sturgeon. In Lake Sturgeon, calcium is utilized to produce defensive dorsal scutes, which are mostly made up of calcium-based minerals, most likely hydroxyapatite, with substantial phosphate and carbonate content [19]. When they are still developing, Lake Sturgeon rely on their scutes for protection [20]. For hatchery-reared Lake Sturgeon, stocking survival is thought to be associated with fish size [21]; fish are therefore reared to a relatively large size (>10 cm total length) in the hatchery prior to release. If Lake Sturgeon develop scutes faster and larger while young, this could help the stocked fingerlings to have better protection when they are released into the river system.

This study examined the growth, ion storage, and scute development of Lake Sturgeon, via three specific hypotheses: (1) ion accumulation in tissues of larvae will be directly correlated with environmental concentrations, (2) the period of active larval growth, measured as the rate of increase of body mass and total length, will stabilize earlier in water with higher calcium compared to lower calcium, and (3) Lake Sturgeon in water with higher calcium levels will develop scutes before Lake Sturgeon in water with lower levels of calcium.

## 2. Materials and Methods

### 2.1. Aquarium System

**Figure 1:**
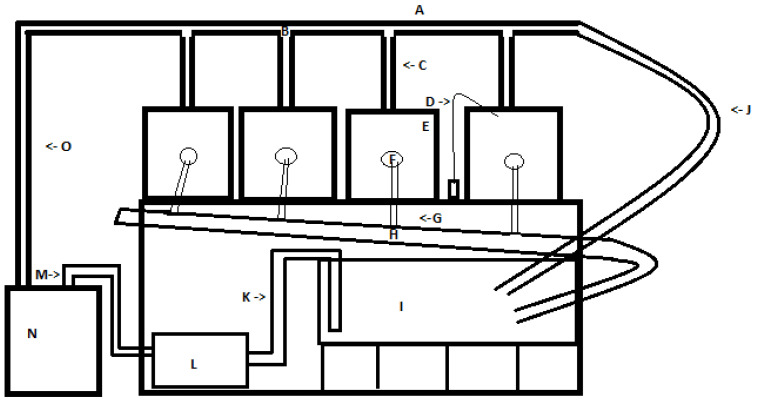
Recirculating water system. (**A**) = upper outflow pipe, (**B**) = water spigot, (**C**) = hose from spigot to tank, (**D**) = air hose, (**E**) = tank, (**F**) = lower output, (**G**) = hose from output to collector pipe, (**H**) = collector pipe, (**I**) = sump, (**J**) = overflow pipe, (**K**) = pipe from sump to water pump, (**L**) = water pump, (**M**) = pipe from pump to chiller, (**N**) = chiller, and (**O**) = side pipe to upper output.

We set up a recirculating aquarium system (Figure 1) to mimic the system in use at the Warm Springs National Fish Hatchery (WSNFH), consisting of four, 38 L tanks (Figure 1E) which drained to a 208 L sump tank (Figure 1I) which was elevated off the ground to allow proper flow to the filter and help with temperature control. Tank water was maintained at 15 ± 1 °C at both the University of West Georgia aquatics research lab (UWG) and WSNFH using a chiller (AquaEuroUSA Max-Chill, Aquacave Inc., Lake Forest, IL, USA) (Figure 1N), and recirculated with an external water pump (Little Giant Pump, Franklin Electric Co., Fort Wayne, IN, USA) (Figure 1L). Two air pumps (Penn-Plax Silent-air XS, Hauppauge, NY, USA) (Figure 1D), and one canister filter (Penn-Plax Cascade 700, Hauppauge, NY, USA) were used to maintain consistently high oxygen levels and low nitrogen levels in the system.

In each 38 L tank, we had an outflow drain from the top of the tank (Figure 1F). The outflow grill was covered with a piece of fine mesh fabric to make sure only water would go down the drain. Water was returned to the upper tanks from the sump using an external pump, connected to the chiller for temperature control. The pipes above the tanks we called the upper outflow, which included not only the water input for the upper tanks, but also a water line for surplus water to bypass the upper tanks and drain into the sump to control pressure and water flow coming out of the upper outflow (Figure 1J). For the lower outflow of water from the four tanks to the sump, half-inch pipes connected each tank to a collection pipe (Figure 1H), which was slanted at an angle to keep water flowing down towards the sump. To connect all the PVC pipes, we used water-safe PVC cement (Oatey rain-R-shine medium blue). Door screening fabric was used to build tank dividers to keep larvae from getting near the overflow drain. We slid the fabric into plastic clips and used aquarium-safe sealant (Marineland Aquarium) to attach the holders into the tank. Before we received Lake Sturgeon embryos, we filled all the tanks with water collected from the Coosa River and ran the recirculating system for 2 weeks to make sure it was working properly and establish the biological filters prior to the introduction of the Lake Sturgeon. Water was tested for appropriate water quality parameters before putting larvae in the system.

Water was collected from the Coosa River (location: 34.380306, −85.123845) by GA-DNR personnel and delivered to UWG using a fish-stocking truck. The water was transferred by pump to four large (>550 L) holding tanks for storage. We then placed a water filter and airline (Penn-Plax Silent-air X5, Hauppauge, NY, USA) in each tank. The water stayed in these holding tanks until it was needed for water changes and to top off the water in the tanks after feedings to keep a consistent total water volume and maintain good water quality.

The room lights were kept off in the lab during the entire study duration. Lab personnel used headlamps on the low setting during feeding or other work near the tanks. Nearby overhead lights were set to a light timer to turn on at 7:30 am every morning and to go off at 9 pm every night. This matched up with the natural sunrise and sunset times for the region in May/June. To mimic their natural environment of light only coming from above the larvae, and to minimize stress, we blacked out the sides of the tanks with black contact paper to keep any light from coming in on the sides of the tanks.

### 2.2. Larval Rearing

Procedures involving live animals used in this study were approved by the Institutional Animal Care and Use Committee of the University of West Georgia (protocol #1201). Lake Sturgeon eggs were obtained from the Wolf River, WI by USFWS personnel. Immediately after transport to WSNFH (Warm Springs, GA, USA), a subset of 700 eggs was transported in coolers to the aquatics lab at UWG and placed in the prepared tanks filled with water collected from the Coosa River. In this way, two subsets of a single cohort were reared from the egg stage in either Coosa River water (at UWG) or under standard hatchery conditions (at WSNFH).

We placed Lake Sturgeon eggs into two egg jars made with two 2 L bottles, 1″ PVC tubing, and aquarium-safe sealant (Marineland Aquarium), following a previously described procedure [22]. We placed half of the eggs in each egg jar. Each egg jar had a water pipe that went down into it, generating a small flow to gently circulate the eggs within the bottom third of the jar. The egg jars were checked multiple times a day to make sure no fungi or dead eggs had accrued. Eggs that were not viable were removed to keep any fungi from developing.

After the Lake Sturgeon hatched out, they exhibited swim-up behavior, and we placed them into submersible breeder boxes with mesh around the inside to keep them from escaping through the drain holes on the sides. Once the Lake Sturgeon began to consume live feed (*Artemia* spp.), they were placed in two larval baskets secured to the inside of the tank above the water line of the main tank (Figure 1E) to ensure water could flow in from the top of the larval holder and then drain to the sump for mechanical and biological filtering. These baskets had six, 0.5’’ holes down the side with mesh covering each hole so water and excess food could flush out but not the larvae, while maintaining a water depth of approximately 4 inches. We would turn the water off when we were feeding the Lake Sturgeon. Out of the four tanks, only two were used to hold larvae at any given time.

We performed a weekly water change of 38 L. We pulled the water from the sump or drained one of the smaller tanks to do a tank cleaning and water change at the same time. We did not clean tanks that had a sturgeon holder in them to make certain we did not disturb the larvae. We then obtained water from the holding tanks to replace the water in the tank system. Commercial testing kits (API) were used regularly to check for nitrogenous compounds such as nitrate, nitrite, or ammonia in the water sample; any evidence of nitrogenous waste buildup would be followed by a water change to keep levels as near to zero as possible.

At 12 days post-hatch (dph), the larvae were offered *Artemia* as the initial feed (Argent Aquaculture Argentamin Gold Grade I Brine shrimp eggs 90% hatch rate). We made a solution of salt-water to hatch the *Artemia* cysts according to the provided recommendations by dissolving 25 g of non-iodized salt and 2 g of sodium bicarbonate into 950 mL of deionized water. The salinity was confirmed as 28–30 ppt using a portable refractometer. Large batches of the saltwater were made every three days. We used a water bottle hatching system to incubate the *Artemia* to feed the Lake Sturgeon. To do this, we placed six water bottles (500 mL each) upside down in a metal holder with the bottoms cut out of the bottles. Each water bottle was filled with 500 mL saltwater and 1.2 g of *Artemia* cysts per jar, so that one hatched jar was sufficient for each larval feeding. Once filled, the bottoms of the bottles that were cut off were placed back on the bottle as a “lid” to reduce evaporation, with small holes in them so they would not build up pressure and pop off. We replaced the bottle caps with a cap that had two out ports: one for air, which also provided circulation of the cysts, and the other to retrieve the hatched *Artemia*. The temperature of the jars was maintained at 28 °C by using two 100 watt bulbs in terrarium reflecting lamps at a set distance from the bottles. A temperature gauge in the bottle was used to monitor the temperature. Hatch out of *Artemia* occurred within 24 h, at which point they were retrieved by turning off the airlines and moving the light towards the cap (bottom) of the bottles. After 20 min or when most of the *Artemia* had moved towards the cap, we collected them from the bottles using the siphon line with a spigot at the end. The *Artemia* were emptied out over a plastic beaker and strained into a fine-mesh net. We cleaned off the saltwater by dipping the net into another plastic beaker filled with water from the tank system. At each feeding time, we evenly distributed the *Artemia* from one entire jar to the larval holders using plastic transfer pipettes. Frozen cubes of baby brine shrimp (Hikari Bio-Pure) were used for supplementary feeding as needed.

We fed the Lake Sturgeon larvae four times a day at 2 am, 8 am, 2 pm, and 9 pm. At each feeding, the Lake Sturgeon were given an entire jar of hatched *Artemia* ranging in volume from 6 mL to 12 mL and allowed to feed to satiation for 1–2 h. In general, the nutritional value of *Artemia* with regard to the ions of interest in this study has been reported as: sodium (2.1–51.1 mg·g^−1^), potassium (0.73–12.7 mg·g^−1^), magnesium (1.05–6.8 mg·g^−1^), calcium (0.2–4.8 mg·g^−1^), and zinc (75–241 µg·g^−1^) [23]. After each feeding event, any leftover food was gently siphoned out of the larval holder using a modified tank gravel cleaner. We measured how much water was removed from the recirculating water system and replaced the same volume of water from the reservoir tanks into the system at the sump.

### 2.3. Water Sampling

We collected 40 mL aliquots of water from the sump, water held in the river water reservoir tanks, and from WSNFH each time Lake Sturgeon samples were taken (Appendix A (Table A1)). We used water testing strips (Tetra Eazy Strips 6-in-1) to do quick testing of the water quality. We also took samples of the water and tested it for ammonia (NH_3_/NH_4_^+^), nitrite (NO_2_^−^), nitrate (NO_3_^−^), and general and carbonate hardness (GH and KH) using commercial testing kits (API). We used a portable pH meter (Cole Parmer 423) accurate to ±0.01 units to test water pH. Following these initial water quality checks, the water samples were stored in a refrigerator at 4 °C for later analysis of ion content (Na^+^, Ca^2+^, Mg^2+^, K^+^, and Zn^2+^) via Inductively Coupled Plasma Optical Emission Spectrometry (ICP:OES).

### 2.4. Larval Sampling

We made a pH-buffered solution of tricanine methanesulfonate (MS-222) in 150 mL water by adding 0.33 g of MS-222 and 0.105 g of sodium bicarbonate. Larvae identified for sampling were quickly transferred using a wide-mouth pipette to 40 mL of water and then 10 mL of the MS-222 stock solution was added to dilute to the final concentration (440 mg/L). All sampled fish were euthanized simultaneously for each sampling point. Prior to taking physical measurements, mortality was confirmed by watching for any movement within a 10 min period after flipping the bottle over. Our first three samples were taken in the first week, after which samples were taken two times a week for two weeks (*n* = 10–12 per sample). After this, samples were taken once a week. We sampled four weeks longer at WSNFH than we did at the UWG lab (Table A1), due to higher mortality at UWG. All Lake Sturgeon had fasted for 12 h before sampling to make sure subsequent analysis represented the tissue of the fish only and did not include any gut content.

Sampling protocols until 11 dph were identical at WSNFH and UWG. Fish were euthanized, measured for total length with an electronic digital caliper to the nearest 0.01 mm, and placed in individual pre-weighed microcentrifuge tubes, and then reweighed to determine the mass of each sampled fish. After the Lake Sturgeon began feeding on *Artemia* (15 dph, Table A1), we began to bring them back to the UWG lab alive to check for any scute development instead of taking measurements at WSNFH. We brought the Lake Sturgeon back in a plastic bag placed in water within a cooler, and continuously aerated with a portable air pump. An ice pack was placed in the bottom of the cooler to keep the temperature below 19 °C.

After doing the initial anatomical measurements, we placed the Lake Sturgeon on a Petri dish and placed the dish on ice to prevent tissue degradation during dissection. While on the ice, the samples were placed under a dissecting stereoscope (Practum sartorius) and the scutes were cut off using a dissecting blade. The scutes were placed in their own pre-weighed tubes and weighed. The fish was also reweighed to confirm no weight was lost during the dissection apart from the removed scutes.

### 2.5. Sample Ion Analysis Using Inductively Coupled Plasma Optical Emission Spectrometry (ICP:OES)

All Lake Sturgeon samples were placed in the freezer at −20 °C until they were processed for ion composition analysis. Samples were pulled out of the freezer at random to prevent sample bias within any given processing batch, and each box with samples was out of the freezer for less than 2 min while all samples that were needed for an individual round of analysis were pulled out to prevent freeze–thaw cycles.

The total mass of each fish was used to calculate how much nitric acid (70% HNO_3_) was needed. For a mass of 0.05 g or under we added 110 µL of nitric acid and scaled the volume by 100 µL increments per additional 0.05 g. After nitric acid was added, the test tubes were vortexed to mix the acid and sample together. The samples were left at room temperature for 24 h to ensure complete digestion of the tissues. During the 24 h, the samples were vortexed twice to aid acid digestion. After the samples were completely dissolved, they were centrifuged at 12,000× *g* for 2 min to pellet any non-soluble components to the bottom of the tube. We removed 100 µL of the sample from the test tube and placed it in a 15 mL test tube with 100 µL of DI water and then added 100 µL of 30% H_2_O_2_ to keep the proteins from precipitating out of the solution. The last step was to add 9.7 mL of 4% nitric acid to the test tube to have a total volume of 10 mL with a final acid concentration of 4.5% by volume.

Natural water samples were prepared for ICP-OES using a simplified procedure similar to the fish sample preparation. First, the sample tube was centrifuged to remove particulates. Then, the sample was stabilized at pH < 2 by combining the sample with HNO_3_ to a final concentration of 5% acid by volume (1 mL 70% HNO_3_ added to 13 mL sample).

We followed EPA method 200.7 for sample preparation and analytical protocols [24]. The ICP:OES instrument used five different concentrations to set a standard curve for each ion of interest ranging from 0.001 to 10.0 mg/L. If necessary, samples were diluted to be within this range then back-calculated to know the final ion concentration by using the dilution factor. Samples were pumped using an autosampler from the 15 mL test tubes into the ICP:OES at approximately 1 mL per minute. We analyzed emission spectra for calcium at two wavelengths (393.366 nm; 317.933 radial and axial), along with magnesium (270.553), potassium (766.490), sodium (589.592), and zinc (213.857). The ICP:OES provided the ion concentration of the sample in mg/L using the calibration standard curve, and these values were then standardized to the total mass of each individual fish (mg/g).

### 2.6. Statistical Analysis

All data are reported as the mean ± SEM (standard error of the mean). The statistical software Sigmaplot was used for all data analysis. The water quality and ionic composition of the two rearing treatments were compared using an unpaired *t*-test. A two-way ANOVA was used to compare between treatment groups for each parameter measured for larval samples (mass, ion concentration) and time as the second factor, as well as any interaction effects. For our post hoc test, we used a Holm–Sidak test. Values below *p* < 0.05 were considered to be significant.

## 3. Results

### 3.1. Water Sample Ion Composition

Water testing indicated that pH (7.3 ± 0.09), NO_2_^−^ (0.15 ± 0.07 ppm), NH_3_ (0.18 ± 0.12 ppm), NO_3_^−^ (15.5 ± 3.90 ppm), GH (104.15 ± 3.05 ppm) and KH (71.6 ± 0.00 ppm) were stable for the collected Coosa River water over the experimental period. Stable conditions also were maintained at the WSNFH. ICP:OES results for the water sampled during the experiment indicated water from the Coosa River and WSNFH water differed significantly for most ions tested (Table 1). WSNFH water had almost double the concentration of calcium found in Coosa River water, while Coosa River water had significantly higher magnesium and sodium levels than WSNFH water. Although it was not included in our initial suite of ions to be analyzed, it was noteworthy that zinc was found to be four times higher in Coosa River water than it was at WSNFH water (Table 1). Potassium was the only ion examined which did not differ between the two types of water.

### 3.2. Whole-Body Ion Concentrations

**Figure 2:**
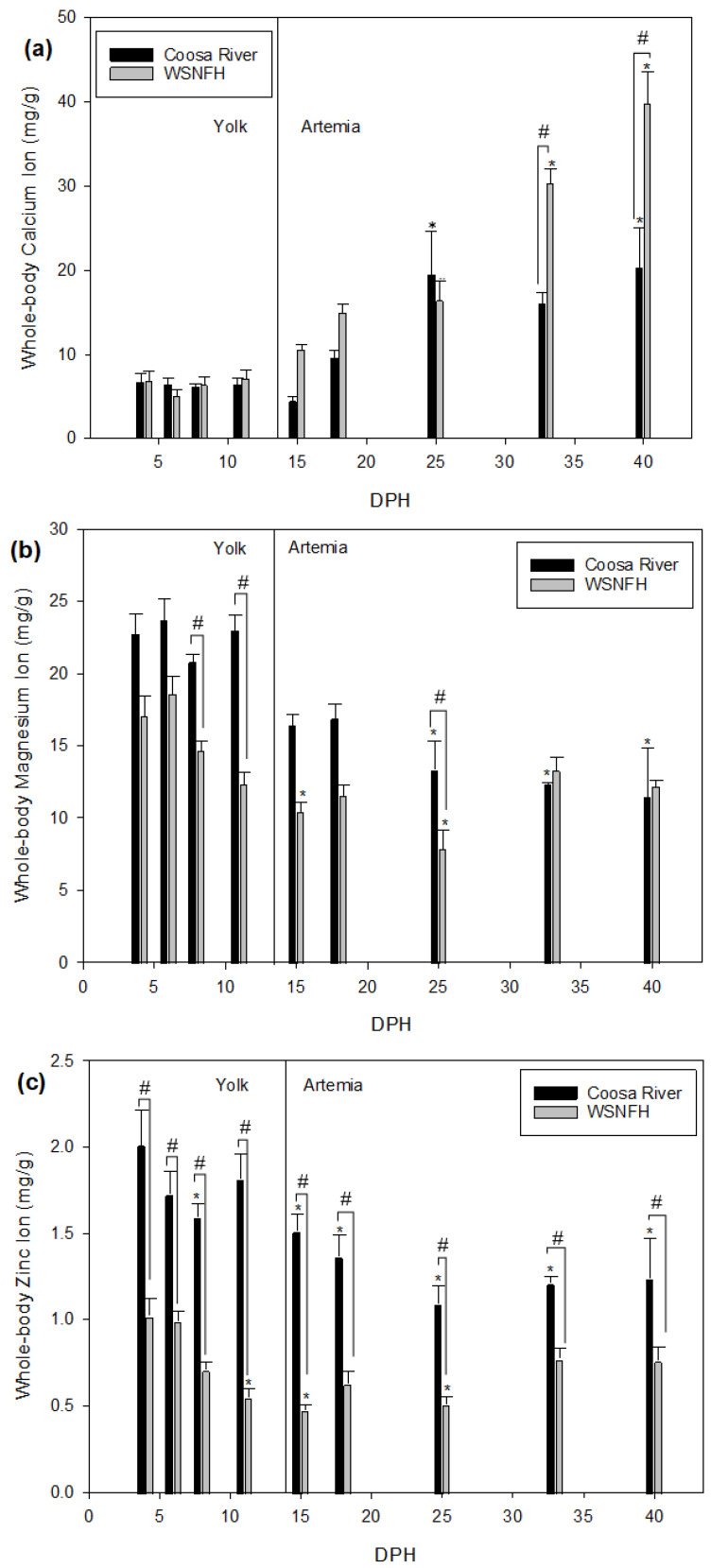
Whole-body ion levels (mg/g) without scutes of (**a**) calcium, (**b**) magnesium, and (**c**) zinc in larval Lake Sturgeon (*n* = 3–9 per sample point). Black bars represent fish reared in Coosa River water, and gray bars represent fish reared at Warm Springs National Fish Hatchery (WSNFH). * = significant difference between the sample point and 4 dph within the treatment. # = significant difference (*p* < 0.05) between treatments for that sample point. Larvae relied on yolk from 0 to 14 dph and were fed live *Artemia* from 15 to 40 dph.

Lake Sturgeon larvae were still dependent on their yolk until 11 dph. *Artemia* was offered starting on 12 dph until 40 dph to Lake Sturgeon in both treatments. All the larvae were consuming *Artemia* by 15 dph. Lake Sturgeon reared in WSNFH water had higher whole-body concentrations of calcium than Lake Sturgeon reared in Coosa River water at the time points after feeding onset (15 dph), although this difference was not statistically significant until 33 dph (Figure 2a). Overall, calcium in Lake Sturgeon larvae reared in Coosa River water and at WSNFH were significantly different (*p* = 0.0006). Compared to the first sample point (4 dph), Lake Sturgeon reared in Coosa River water had significantly increased calcium starting at 25 dph. This difference occurred later for Lake Sturgeon reared in WSNFH water, where calcium increased significantly from the first sample point starting at 33 dph. Results of a two-way ANOVA showed a significant interaction effect between time and treatment with regard to calcium (*p* < 0.0001), so paired comparisons across time and between treatments should be considered with this in mind.

Lake Sturgeon reared in Coosa River water have higher levels of magnesium in their body compared to larvae reared in WSNFH water in every sample point except for 33 and 40 dph (Figure 2b), although these differences were only significant at 8, 11, and 25 dph. All samples in either treatment that were significantly different from the first sample point (4 dph) occurred after feeding onset (15–33 dph). A two-way ANOVA indicated that there was a significant difference between treatments (*p* < 0.0001) and time (*p* < 0.0001), as well as a significant interaction effect (*p* = 0.0011). Larvae dependent on yolk (4–11 dph) had higher whole-body magnesium levels than for fish feeding on *Artemia* (15–40 dph), and juveniles at the later stages have a smaller difference between treatments. Thus, it appears that levels of magnesium may be associated with feeding state more than they are dependent on water chemistry differences.

Fish reared in Coosa River water had significantly more zinc in the body compared to WSNFH (Figure 2c), mirroring the concentration differences observed in the ambient water (Table 1). From 4 to 25 dph, larvae reared in Coosa River water had at least twice the amount of zinc in their tissues than larvae reared at WSNFH. At 11 and 15 dph, the difference was three times the amount of zinc than larvae reared in WSNFH water. The later sample points show a decrease in the amounts of zinc found in the bodies of both treatments primarily after feeding onset, particularly in fish exposed to Coosa River water. Over developmental time, a two-way ANOVA (*p* < 0.0001 dph, *p* < 0.0001 treatment) indicated that there was a significant change in Lake Ssturgeon reared in Coosa River water compared to Lake Sturgeon reared in WSNFH, and these main effects had significant interaction (*p* = 0.0012).

**Figure 3:**
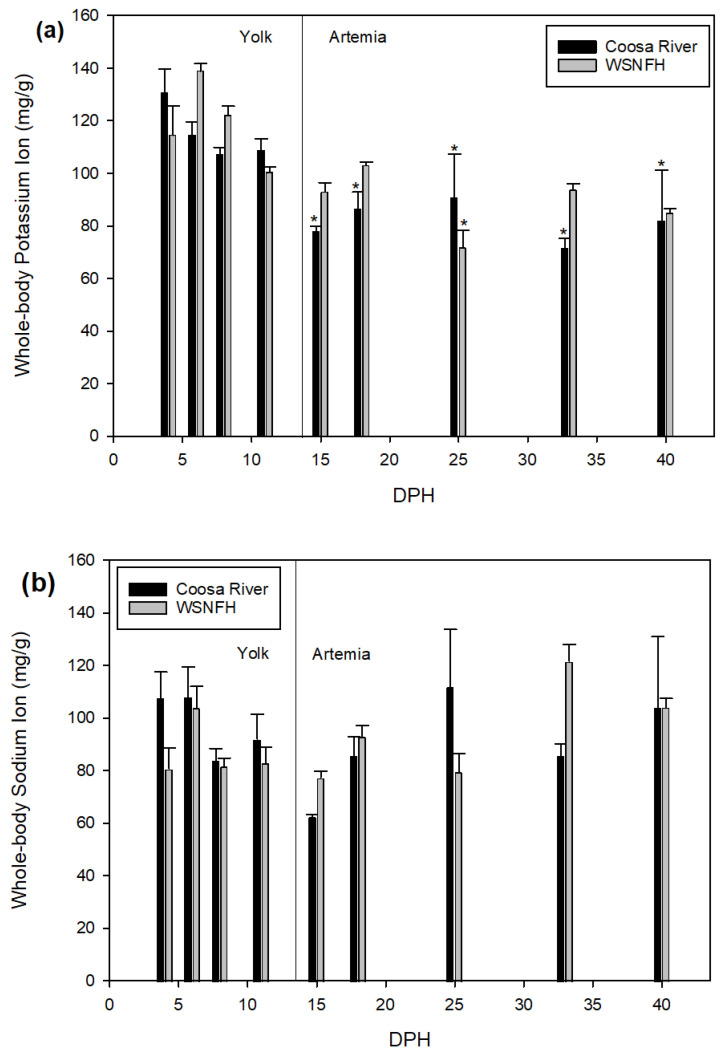
Whole-body ion levels (mg/g) without scutes of (**a**) potassium and (**b**) sodium in larval Lake Sturgeon (*n* = 3–9 per sample point). Black bars represent fish reared in Coosa River water and gray bars represent fish reared at the Warm Springs National Fish Hatchery (WSNFH). * = significant difference between the sample point and 4 dph within the treatment. Larvae relied on yolk from 0 to 14 dph and were fed live *Artemia* from 15 to 40 dph.

Lake Sturgeon reared in Coosa River water had similar potassium levels compared to fish reared at WSNFH (*p* = 0.1633) (Figure 3a). All sample points in Coosa River or WSNFH water that were significant when compared to the first sample point 4 dph occurred after the transition to live feed (*p* < 0.0001) and mostly in Coosa River water, with larvae at WSNFH displaying a significant difference only at 25 dph. There was no interaction effect between developmental time and rearing environment (*p* = 0.0601). Overall, there was no significant difference between Lake Sturgeon reared in Coosa River water and WSFNH water regarding sodium (*p* = 0.0939 dph, *p* = 0.7093 treatment, *p* = 0.0921 interaction) (Figure 3b).

### 3.3. Larval Growth

**Figure 4:**
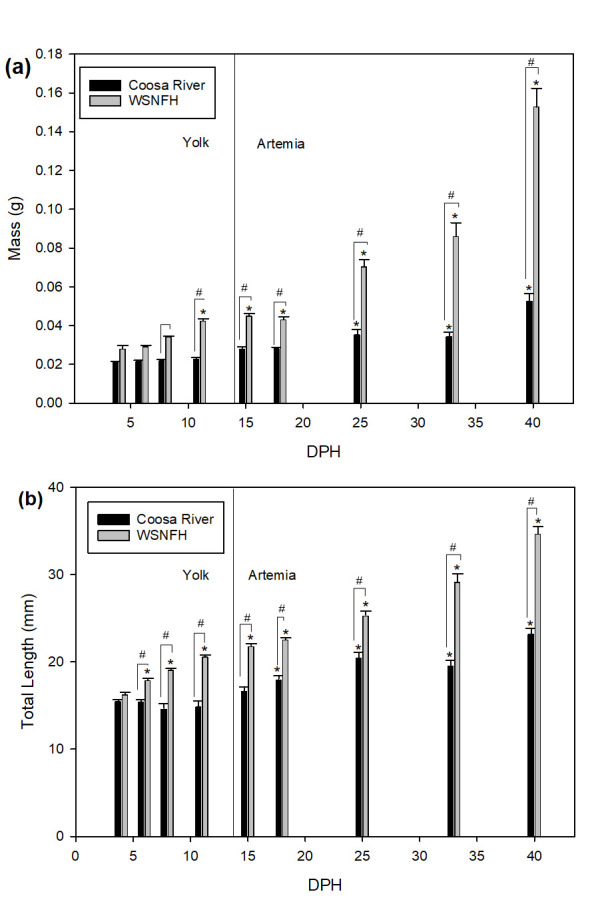
(**a**) Total mass without scutes and (**b**) total length of larval Lake Sturgeon (*n* = 10–12 per sample point; 15 days post-hatch (dph), *n* = 3). Black bars represent fish reared in Coosa River water and gray bars represent fish reared at the Warm Springs National Fish Hatchery (WSNFH). * = significant difference between sample point and 4 dph within the treatment. # = significant difference between treatments for that sample point. Larvae relied on yolk from 0 to 14 dph and were fed live *Artemia* from 15 to 40 dph.

There was a significant difference between Lake Sturgeon reared in Coosa River water and in WSNFH water regarding total mass without scutes (Kruskal–Wallis one-way ANOVA on Ranks: *p* < 0.0001 (time and treatment)). Lake Sturgeon reared in Coosa River water at 25, 33, and 40 dph were significantly larger than at the first sample point 4 dph (Figure 4a), while this was true earlier for Lake Sturgeon reared in WSNFH water, which were significantly larger starting at 11 dph. Overall, WSNFH Lake Sturgeon weighed more than Lake Sturgeon in Coosa River water throughout the experiment (starting at 8 dph).

The total length of larvae reared in Coosa River versus WSNFH water showed the same trend as total mass, with WSNFH fish being longer at every sample point after 6 dph (Figure 4b), based on a one-way ANOVA on ranks (*p* < 0.0001 (time and treatment)). This was consistent with changes in total length over time, where WSNFH Lake Sturgeon were significantly different from the first sample point, 4 dph, starting only 2 days later (6 dph). Lake Sturgeon reared in Coosa River water, in contrast, were not significantly longer from the first sample point until 18 dph. Fish reared in Coosa River water did not significantly increase in mass or total length until after feeding onset (15 dph).

### 3.4. Scute Development

**Figure 5:**
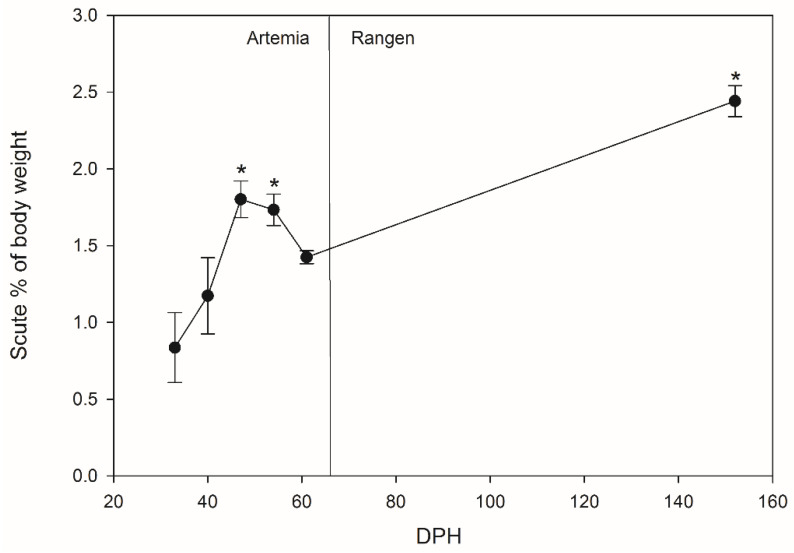
Scute mass as % of body weight in Lake Sturgeon at Warm Springs National Fish Hatchery (WSNFH) (*n* = 10–12 per sample point). * indicates a significant difference between the sample point and 33 dph. Fish were fed live *Artemia* from 15 to 61 dph, and commercial chow (Rangen) from 62 to 152 dph. Kruskal–Wallis one-way ANOVA on Ranks: *p* < 0.0001.

Scutes were only able to be collected from Lake Sturgeon reared at WSNFH, as fish reared in Coosa River water did not develop these structures prior to the end of the experimental period (40 dph, Table A1). There was a significant increase in the proportion of body mass accounted for by scutes at 47, 54, and 152 dph, compared to the first point scutes were observed (33 dph) (Figure 5). We observed a rapid increase in the scute percentage of the body until 47 dph, after which the rate of increase was slower, and scutes represent a substantially larger percentage of total body mass at the end of the experiment (152 dph). There was a small reduction at 61 dph, which was likely due to the introduction of pellets (Rangen) as the primary feed, combined with a chiller failure which increased the temperature an unknown amount for a few hours until the system was repaired the following morning.

## 4. Discussion

### 4.1. Whole-Body Ion Levels

Lake Sturgeon are typically found in lakes that have low levels of calcium [13], which was consistent with our Coosa River water (Table 1). WSNFH water had almost double the amount of calcium available than Coosa River water (Coosa River: 14.02 mg/L vs. WSNFH: 27.84 mg/L, Table 1). Whole-body calcium concentrations of Lake Sturgeon in Coosa River water and WSNFH water were about the same from 4 to 11 dph. During this time, Lake Sturgeon in both treatments were still dependent on calcium from their yolk. Differences later in development (>33 dph) between the calcium levels of Lake Sturgeon reared in Coosa River water and WSNFH water samples are present in the tissues, independent of any sequestration of calcium that may occur into the scutes (Figure 2a). Interestingly, mass-specific calcium concentrations were higher in WSNFH larvae at 33 and 40 dph; therefore, the treatment with the fastest scute development also showed increased body tissue levels, indicating that calcium uptake may be even more pronounced in this group. After the onset of exogenous feeding a difference between the two different calcium levels demonstrated in the whole-body ion composition becomes evident (Figure 2a). It is important to note that all feed was digested before sampling, and both treatments received the same type of food at the same times, so even post-feeding, water chemistry appears to be influencing whole-body ions. We do see a significant change over time in Lake Sturgeon reared in both water treatments. This can be attributed to absorption from the water, since that would be the main source that differs between the two treatments. Lake Sturgeon in both treatment groups demonstrate a significant difference at 25 dph and later. This may be related to the developmental stage, as Lake Sturgeon tend to require more calcium as they develop and mature from the larval to juvenile to adult stages [25]. In adults, this culminates in the relationship between oogenesis and regulation of circulating calcium levels [25]. While the Lake Sturgeon in the present study are not near their reproductive age by any means, calcium will need to start being absorbed at a young age to develop their scutes, which are not bone, but are ossified structures [26]. The increase in weight-specific whole-body calcium coincides with the initial development of the calcium rich scutes (33 dph for WSNFH, Figure 5).

Lake Sturgeon reared in Coosa River water had higher overall levels of magnesium in their bodies compared to Lake Sturgeon reared in WSNFH water prior to the onset of exogenous feeding. This likely is associated with the higher levels of magnesium in the Coosa River compared to WSNFH water. This could mean that if more magnesium is available the Lake Sturgeon will absorb it, and implies they rely primarily on branchial uptake for this mineral. When juvenile Persian Sturgeon (*Acipenser persicus*) were given higher magnesium diets, they did not absorb any more magnesium than Persian Sturgeon given a standard magnesium diet [27], supporting the idea that sturgeon rely on branchial absorption as the main magnesium uptake pathway. For both treatments, the levels of magnesium in the yolk stage are higher than during the *Artemia*-feeding stage (15–40 dph) (Figure 2b). The clear decrease in mass-specific whole-body magnesium levels in this study shows that sturgeon may rely on their environment (water) more to help maintain magnesium levels, rather than on availability in the feed, and that uptake capacity declines with growth. Magnesium also is associated with the transport of calcium, providing further evidence that uptake at the gill is the most important factor for uptake of these divalent cations.

The Coosa River water has over 3.5 times more zinc than WSNFH has in its water (Table 1). That would help explain why most Lake Sturgeon tissue samples have at least double the zinc when reared in Coosa River water compared to WSNFH. When there are higher levels of zinc in the body of a fish, it can stay in their system and build up; this could be why when the Lake Sturgeon were placed in water that has higher levels of zinc they also contained higher levels of zinc [28,29]. Having a buildup of zinc in a Lake Sturgeons’ system could lead to stunting of growth [14], which we did see in Lake Sturgeon that were placed in higher levels of zinc (Figure 4). Zinc also has been linked to negative impacts on reproduction in teleost fishes [29]. Given the concentrations of zinc measured from the Coosa River of the Lake Sturgeon in this study (Table 1), future work investigating whether this is impacting natural recruitment may be an important line of research. Furthermore, zinc and calcium can interact with one another. Zinc is a competitive inhibitor to calcium influx, while calcium is the same for zinc influx. It has been shown that calcium goes through the gill epithelium in the same location as zinc [30]. Relative water hardness (i.e., calcium concentration) compared to zinc is known to be protective against toxicity. In this study, the combination of high calcium and low zinc at WSNFH may have reduced chronic effects of zinc uptake, although no substantial differences in mortality were observed during the first 46 days post-hatch. Trace metal accumulation in chub has been demonstrated to be linked to growth stage, and variable across ontogenetic feed transitions. In chub, juveniles had higher tissue concentrations of zinc than the later life stages, suggesting that in at least some freshwater fishes, zinc may bioaccumulate differently than macrominerals, including those considered in the present study (i.e., Ca^2+^, Na^+^, K^+^, Mg^2+^) [31]. Whether naturally recruited larvae are at greater risk of zinc toxicity due to their size, diet, and/or the low hardness buffering capacity of Coosa River water is a question that should be addressed by future studies.

Lake Sturgeon reared in Coosa River water and WSNFH water were not significantly different in tissue levels of potassium (Figure 3a), corresponding with the fact that the potassium in the water was not significantly different between treatments (Table 1). Depending on the sampling time, Lake Sturgeon from either Coosa River or WSNFH treatments had higher levels of potassium in the body, but no discernible pattern was evident over the study duration.

Green Sturgeon (*Acipenser medirostris*) and Atlantic Sturgeon (*Acipenser oxyrinchus*), which are anadromous and live in saltwater at later stages of their life, increase their number of mitochondria-rich cells (MRCs) when placed in higher levels of sodium [32,33,34,35]. Although Lake Sturgeon are in the same family as Green and Atlantic Sturgeon, they do have different life histories and are non-migratory. However, they may demonstrate similar cellular responses to increased ambient sodium. If so, we would expect to see higher levels of sodium in the tissues of Lake Sturgeon reared in water with higher levels of sodium. The levels of sodium found in our treatments were significantly higher in Coosa River water (Table 1), but the tissue samples from Lake Sturgeon exposed to this environment were not significantly different (Figure 3b). The sodium levels between Coosa River water and WSNFH water varied at each sample point, with no clear trend as to which treatment had higher total body sodium levels. To our knowledge, sodium uptake in larval or juvenile Lake Sturgeon has not been investigated, and this would need to be further studied to see if Lake Sturgeon have similar regulation of sodium transport pathways as other sturgeon species.

### 4.2. Larval Growth and Development

Lake Sturgeon reared in WSNFH water weighed more than Lake Sturgeon reared in Coosa River water at every sample point from 8 to 40 dph, and fish reared at WSNFH were significantly larger than fish in Coosa River water much earlier in development (11 dph vs. 25 dph). Nearly the same results were reflected in the total length of the sampled animals (Figure 4). The Lake Sturgeon in both treatments were still feeding off their yolk until 15 dph, thus differences in growth between these treatments, which are equivalent in diet and developmental pace, may be attributed to the water ion levels. WSNFH water and Coosa River water were both kept at the same temperature range of 15 ± 1 °C. There is a significant difference in the yolk stage at 8 and 11 dph for mass, and 6, 8, 11 for total length, and we attribute this difference to the ambient ion levels resulting in differential availability to Lake Sturgeon reared in each treatment. Looking at the whole-body ions of pre-feeding larvae (8 and 11 dph), we see that there is a trend that Lake Sturgeon in water relatively high in zinc, magnesium, and sodium have significantly lower overall growth. There could be a connection between higher levels of these ions and reduced growth rates of Lake Sturgeon.

We see that as time goes on, scutes become a higher percentage of the total body mass of a Lake Sturgeon. We do see a reduction in the body percentage of scutes at 61 dph; we attribute this decline to the chiller going out at this point in the experiment, and there was also high mortality at WSNFH around this time, so the fish may have been in a relatively poor condition compared to the other sample points. The next step of this project would be to look at the scutes and determine how much calcium is found in scutes. We would predict that the collected scute samples will have higher amounts of calcium the larger the Lake Sturgeon get. Since scutes are mostly calcium and the Coosa River water has lower levels of calcium, this could have delayed the development of scutes and the overall growth in Lake Sturgeon reared in Coosa River water.

## 5. Conclusions

There were many significant differences between concentrations of cations in Coosa River and WSNFH water. We also see significant differences in the total body ions of larval Lake Sturgeon reared in these waters, as well as total length and mass. This shows that when Lake Sturgeon are reared in waters that differ in ionic composition, it can affect the ion concentrations found in their body, as well as influencing their growth and development. This effect is based on ambient environmental conditions, and presumably changes in branchial uptake, rather than gastrointestinal absorption from the diet, which was controlled for in this study. These comparisons provide insight into how hatchery-reared individuals might differ from naturally recruited larvae and can help inform management decisions regarding the conservation aquaculture of this species.

## Figures and Tables

**Table 1:** Ion concentrations (mg/L) measured by Inductively Coupled Plasma Optical Emission spectrometry (ICP:OES) in Coosa River water and WSNFH water (*n* = 11–14). * indicates a significant difference between treatments (unpaired *t*-test, two-tailed, *p* < 0.0001).

Ion	Coosa River Water (mg/L)	WSNFH (mg/L)	Significance
Calcium	14.02 ± 0.24	27.84 ± 1.44	*
Magnesium	3.59 ± 0.06	2.34 ± 0.19	*
Potassium	1.59 ± 0.06	1.62 ± 0.16	
Zinc	0.13 ± 0.02	0.03 ± 0.02	*
Sodium	4.58 ± 0.27	4.21 ± 1.16	*

## Data Availability

The data presented in this study are available on request from the corresponding author.

## Appendix A

**Table A1:** Lake sturgeon sampling days and feeding schedule. Samples (*n* = 10–12) were taken at each listed time given in days post-hatch (dph) from each water treatment. Starting at 47 dph, fish were sampled from Warm Springs National Fish Hatchery only.

Sampling Day (dph)	Feeding
4	Yolk
6	Yolk
8	Yolk
11	Yolk
15 (2 weeks)	*Artemia*
18	*Artemia*
25	*Artemia*
33 (4 weeks)	*Artemia*
40 (5 weeks)	*Artemia*
47 (6 weeks)	*Artemia*
54 (7 weeks)	*Artemia*
61 (8 weeks)	*Artemia*
152 (2l weeks)	Rangen
